# Influence of Short Glass Fibre Reinforcement on Mechanical Properties of 3D Printed ABS-Based Polymer Composites

**DOI:** 10.3390/polym14061182

**Published:** 2022-03-16

**Authors:** Mohan Kumar H. R., Maha Gundappa M. Benal, Pradeep Kumar G. S., Vijay Tambrallimath, Geetha H.R., T. M. Yunus Khan, Ali A. Rajhi, Maughal Ahmed Ali Baig

**Affiliations:** 1 Department of Mechanical Engineering, Government Engineering College, Kushalnagar 571234, India; mmbenal@gmail.com (M.G.M.B.); geethagmb@rediffmail.com (G.H.R.); 2 Department of Mechanical and Automobile Engineering, CHRIST (Deemed to Be University), Bangalore 560029, India; pradeepgs.87@gmail.com; 3 Department of Automobile Engineering, Dayananda Sagar College of Engineering, Bangalore 560078, India; 4 Research Center for Advanced Materials Science (RCAMS), King Khalid University, P.O. Box 9004, Abha 61413, Saudi Arabia; 5 Department of Mechanical Engineering, College of Engineering, King Khalid University, P.O. Box 394, Abha 61413, Saudi Arabia; arajhi@kku.edu.sa; 6 Department of Mechanical Engineering, CMR Technical Campus, Hyderabad 501401, India; mabaig09@gmail.com

**Keywords:** short glass fibre, fused deposition modelling, twin screw extrusion, polymer composite

## Abstract

One of the most promising and widely used additive manufacturing technologies, fused deposition modelling (FDM), is based on material extrusion and is most commonly used for producing thermoplastic parts for functional applications with the objectives of low cost, minimal waste and ease of material conversion. Considering that pure thermoplastic materials have a significantly poor mechanical performance, it is necessary to enhance the mechanical properties of thermoplastic parts generated using FDM technology. One of the conceivable techniques is to incorporate reinforcing materials such as short glass fibre (SGF) into the thermoplastic matrix in order to produce a polymer composite that can be used in engineering applications, such as structural applications. The morphological and mechanical properties of SGF (short glass fibre) reinforced ABS- (Acrylonitrile Butadiene Styrene) based polymer composites created via the method of FDM (fused deposition modelling) were investigated in this work. Properties were evaluated at three different weight percentages (0, 15 and 30 wt%). The composite filaments were developed using the process of twin screw extrusion. The comparison was made between ABS + SGF (short glass fibre) composites and pure ABS of mechanical properties that include surface roughness, tensile strength and low-velocity impact. The tests were carried out to analyze the properties as per ASTM standards. It has been found that the impact strength and tensile strength show an improvement in glass fibre inclusion; moreover, alongside the direction of build, the surface roughness had been reduced. The studies also focused on studying the dispersion characters of SGF in ABS matrix and its impact on the properties. Strength and modulus of SGF reinforced ABS composite has been significantly improved along with reduction of ductility. A 57% increase in tensile strength has been noted for 30 wt% addition of SGF to ABS in comparison to pure ABS. It was also interesting to note the reduction in surface roughness with every incremental addition of SGF to ABS. A 40% reduction in surface roughness has been observed with a 30 wt% addition of SGF to ABS in comparison to pure ABS.

## 1. Introduction

Particularly when compared to the conventional metallic materials, the composites of FRP (fibre reinforced polymer) possess a better capacity of anti-aging, better wear preventing qualities, as well as a high strength-to-weight ratio [1]. In a wide range of industries, the composites of FRP offer tremendous performance and are also lightweight. Thermoplastics, as well as thermosetting, are found in the matrix of FRP composites. Injection moulding/extrusion are the methods for manufacturing and processing short and long FRPs, whilst pultrusion, winding, impregnating, as well as moulding are used to process the continuous fibre-reinforced plastic of other types. Traditional methods for constructing the components of fibre-reinforced plastic are time-consuming, and the process is also complicated; thus, achieving intricate structures is challenging due to the high viscosity required for infusion in the course of wet-out [2,3]. To create products from CAD models, 3D printing is defined as a technique for combining materials [4]. Multipurpose products at a lower cost have often been produced by 3D printing, and it has become popular in recent years to employ this technology to build better products. 3D printing, whether for micrometre solutions or monolithic structure, has proven to be advantageous [5].

The production of an ultimate product by the layer-by-layer deposition procedure is what 3D printing is built on; indeed, faster pace, reduced material waste, higher precision are the significant benefits of this technology compared to conventional prototype techniques. Furthermore, no additional tools are required in a 3D printer, and it has no restrictions on the product’s shape, even when it is highly complicated. FDM (Fused deposition modelling) or FFF (Fused filament fabrication), Laminated object manufacturing (LOM), Stereolithography (SLA), Digital Light Processing (DLP) as well as Selective Laser Sintering (SLS), are all examples of 3D printers that use different technology. The most extensively employed 3D printing technology is the FDM method [6,7]. From medical applications to the aerospace industries as well as automotive, 3D printing is finding an increasing and numerous amount of uses. Along with the different innovations in materials, such as wood or those which are metal-based, which are continually being introduced in the market, 3D printers are becoming more affordable day by day [8,9,10,11]. A CAD file (computer-aided design) is used to begin the printing procedure of an object with the FDM printer; from a coil, the unwinding of plastic filament is done as well as inserted via an extrusion nozzle to produce the object. The filament is melted by the nozzle, which extrudes the melted material onto a foundation known as a bed or build platform. A computer controls the platform as well as a nozzle, and transforms the shape of an object into the axes of x, y, z [12]. Currently, for FDM-based 3D printers, thermoplastic filaments are the principal feedstock. A combination of two thermoplastics, PA (Polyamide), ABS (Acrylonitrile Butadiene Styrene), Polycarbonate (PC), as well as Polylactic Acid (PLA) have all been utilised for this purpose. In 3D printing, and specifically, that which is FDM-based, the Acrylonitrile Butadiene Styrene is the most often used material [12,13,14]. Researchers are attempting to replace traditional moulding processes with 3D printers due to the multiple advantages of such printers against conventional methods of moulding; however, 3D printers are restricted in the type of material they can be fed, which is a key stumbling block to their widespread usage.

As for the manufacturing of the polymer composite, the use of 3D Printing technologies has shown significant economic and timescale advantages, whilst at the same time also delivering various additional benefits, such as rapid iteration, limitless freedom for designers, and being almost irrespective of the complexity of parts; moreover, it allows for lightweight tooling to be produced, which within industrial facilities simplifies the logistics of storage as well as transportation. The composite 3D printer’s improvisation advancements have fuelled the rise in preblended materials and fillers to create different capabilities as well as properties. For improving polymer characteristics, fibre reinforcement appears to be an alluring filler [4,14,15].

Gurrala and Regalla [16] carried out a comparative study between the band strength of filaments developed through FDM. Experimental and mathematical models were developed to analyze the same. The FDM part strength is mostly attributable to neck growth between the filament, intra-layer as well as interlayer bonding, as evidenced by the photomicrographs of the fracture surface taken using a SEM (Scanning electron microscope) and the agreement between the ultimate tensile load of experimental as well as theoretical; it also suggests the availability of time to the filaments as well as the total amount of time, which is just enough to allow them to develop necks and is not sufficient to entirely consolidate. The study did not give propose the justification of strength loss in build direction and transverse direction.

In the process of FDM (fused deposition modelling), Sun et al. [17] have researched the mechanisms that monitor the bonding of extruded polymer filaments. Thermal activity causes bonding phenomena, which dictate the prototypes’ mechanical properties as well as integrity. The study evaluated the quality of the bond by studying changes in mesostructured and also by measuring the level of healing accomplished between neighbouring polymer filaments at the interface. For specimens, the temperature profiles have been measured experimentally, which were made in various situations of processing, whilst the impacts on mechanical characteristics as well as on mesostructures have been seen. Based on the extruded polymer filaments, estimations of the level of bonding accomplished in the course of the process of the filament deposition have been made simultaneously with the experimental study. Mesostructures, cooling temperature profile, and the total bond strength quality between filaments were strongly influenced by the approach of fabrication, envelope temperature, as well as differences in coefficient of convection, according to experimental results. Bond formation was discovered to be affected by the sintering phenomena; however, when the temperature of the filament had been over the critical sintering temperature, it was only for an extremely short time; or else, it was discovered that the creep deformation to govern the alterations in the mesostructure.

WeiZhang et. al. [18] assessed the printed wires of Acrylonitrile Butadiene Styrene’s (ABS) interfacial bonding strength, and also evaluated interfacial bonding strength of short carbon fibre reinforced ABS (CFABS), CNTABS (carbon nanotube reinforced ABS) samples made using the method of FDM. They employed ±45° specimens for the method of double notch shear test, as well as for an in-plane tensile shear test. To the specimen of CNTABS, the CFABS specimen’s in-plane tensile shear strength was almost comparable, and when compared to the ABS specimen, both of these values were higher. For each of the three printing speeds investigated, the CFABS specimens’ in-plane tensile shear strength is greater, meaning that the double notch shear strengths are smaller. As the layer thickness as well as the printing speed increase, the CFABS specimens’ shear strengths also decrease. Compared to the specimens of CNTABS as well as ABS, the CFABS specimens had the most porosity at corresponding raster orientation, specifically for the specimens of ±45°, according to an X-ray micro-computed tomography investigation. The CFABS specimens’ most common dominating modes of failure include an interfacial debonding of fibre matrix, fibre pull-out as well as matrix fracture.

Oscaneiro et al. [19] studied the final product regarding the impacts of the parameters such as glass fibre properties, infill degree percentage, printing orientation as well as thickness layer, and also made a comparison to the method of injection moulding that had been presented. They discovered that introducing 30% of glass fibres increased the modulus and strength of polypropylene by 30% and 40%, respectively. The test specimens, are created by 3D printing; their mechanical properties were 30% lesser when compared those made by injection moulding.

Using an FDM-based printer, Ning et al. investigated the thermoplastic ABS composites usages by varying proportions of carbon fibre, which is included in different sizes. Research findings indicated that the addition of carbon fibre boosted the Young’s modulus and tensile strength of the plastic material, and when compared to the specimens of pure plastic, the material which is of modified had lower yield strength and lower ductility. Furthermore, compared to those containing (100 µm) shorter carbon fibres, CFRP composite specimens enhanced with 150 µm of longer carbon fibres had greater Young’s modulus and tensile strength but lesser ductility as well as toughness [5].

Using the FDM method, Zhang et al. [20] examined fibre-reinforced ABS usage. To boost the strength of the material, the glass fibre had been utilised, but at the same time it also decreased its flexibility. LLDPE had been employed to optimise both flexibility as well as toughness simultaneously; it thus resulted in separation of phase, which changed the filaments’ appearance. Hydrogenated Buna-N had been taken into consideration to address this issue, which resulted in much-enhanced composite properties.

In their research, Kumal et al. [21] looked at the effect of build orientation, platform as well as barrel temperatures, raster angle as well as contours count upon tensile characteristics with regard to elongation percentage, and UTS (ultimate tensile strength) of EVA (ethylene vinyl acetate). Raster angle and barrel temperature had the most influence on UTS (ultimate tensile strength) which is revealed by the results. Moreover, higher and lower UTS values were caused by an enhanced raster angle and barrel temperature, accordingly. Furthermore, the elongation percentage was found to be affected inversely as well as directly by the contours counts and barrel temperature, accordingly. Dimensional stability, toughness, impact resistance, and a smooth surface finish all contribute to ABS’ relevance in industry and science. It is possible to adopt ABS in FDM process because of the material’s ease of flow.

It is generally agreed that there are three steps in the standard production process for fibre-reinforced composites. To begin, prepreg is mostly manufactured using deposition and dipping techniques. Then, using procedures such as spreading moulding, pull-extrusion moulding, winding moulding, and others, it is possible to make composite parts with simple shapes; after this, composite pieces are prepared through machining, assembling, gluing, and other methods. A lengthy production cycle is required for the typical resin-based composite members due to the complexity of the moulding process and the need for secondary processing. The majority of custom moulds have a high cost and just a limited amount of complexity in the pieces. These drawbacks restrict the use of composite materials in a variety of applications. When compared to the traditional approach, 3D printing technology offers a number of significant advantages: no mould is required for this procedure, and it can be made fast and without further processing in a single step; it is theoretically conceivable to manufacture a structure of any shape with this method. At the moment, this may be broadly classified into three types of technologies: Stereo Lithography Apparatus (SLA), Laminated Object Manufacturing (LOM), FDM, and additional technologies, depending on the process flow used. In comparison to other 3D printing technologies, FDM has emerged as a popular research direction in composite 3D printing technology due to its advantages in terms of high material utilisation rate, clean and safe process, rapid forming speed, and low cost, among other things [22,23,24].

In applications requiring a high degree of flexibility, fibreglass is better suited to deliver a higher ultimate breaking point than carbon fibre or other materials. Fibreglass is more adapted to high flex patterns, whereas other fibres have a narrow flex window. Because of its capacity to stretch further without breaking, fibreglass is generally considered a ‘tougher’ material than carbon fibre and Kevlar. Carbon’s remarkable stiffness makes it less resistant to certain abuses than fibreglass. Long strands of carbon fibre are complicated and expensive to produce, whereas fibreglass techniques are simpler. As a result, fibreglass is significantly cheaper than carbon fibre.

There are still significant challenges in 3D printing fibre reinforced polymer composites. In addition to some process-specific limitations, only a few different materials are currently available for fibre reinforced 3D printing, limiting application areas and design flexibility. The addition of (short) fibres to the printing filament increases the stiffness of the part, but the increase in strength is still limited because fibre pull-out may occur prior to fibre breakage. Furthermore, current printing techniques and material options result in significant gaps in finished parts, which reduce the available strength of composites. There is little information available on the mechanical behaviour of short fibre reinforced polymer composites.

The purpose of this article is to discuss the effect of short glass fibre content on the quality and mechanical properties of ABS parts. SGF–ABS composites were successfully prepared and used as FDM feedstocks in this research. To evaluate the FDM technique’s mechanical properties, SGF (short glass fibre) reinforced ABS composites with varying fibre loadings were developed using FDM; the effect of fibre loading on the impact strength and tensile strength modulus of final printed products was also investigated.

## 2. Experimental Procedure

**Figure 1:**
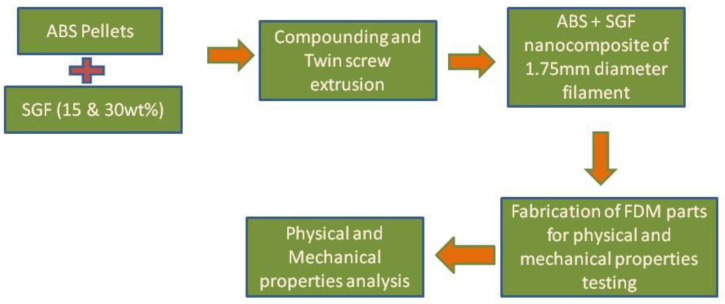
Process flowchart.

Figure 1 shows the process flowchart of the experiment conducted. 

### 2.1. Raw Materials

**Figure 2:**
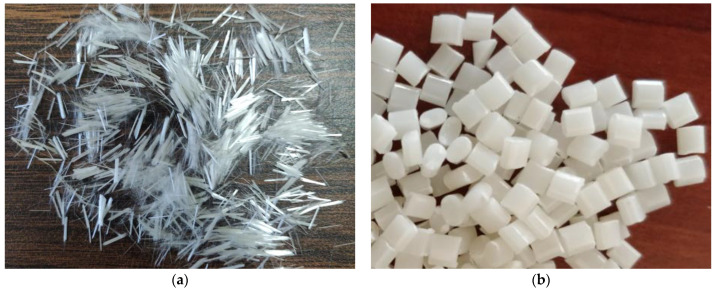
Photograph of as received (**a**) short glass fibre and (**b**) ABS pellets.

**Figure 3:**
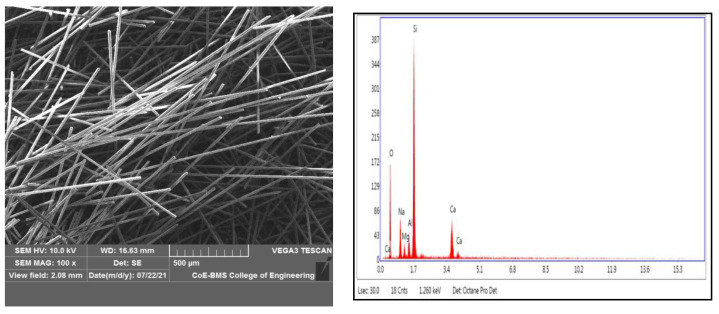
SEM and EDAX of as received short glass fibre.

ABS pellets were used as matrix material, procured from M/s GLS polymers, Bangalore, INDIA. Short glass fibres used as reinforcement were supplied by the M/s Tespo International, Bangalore, INDIA. Figure 1 shows the process flowchart for the present work. The short glass fibre and ABS pellets are shown in Figure 2a,b, respectively. Figure 3 shows the SEM and EDAX of as received short glass fibre.

**Figure 4:**
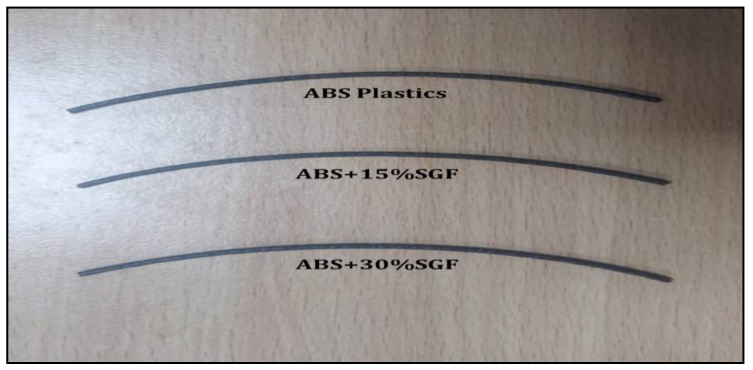
Photograph of ABS and ABS + SGF filament developed by twin screw extrusion.

Removal of moisture plays a prominent role in improvisation of functionality and smoothing the flow process. ABS pellets were dried for a period of 2 h at a temperature of 120 °C. Once the process of drying was completed, these dried pellets were subjected to the compounding process. Short glass fibres were measured in definite quantities and added to these dried pellets before being fed into the compounding machine. Compounded pellets were subjected to a twin screw extrusion process. For the extrusion of 1.75 mm diameter filament, a single screw double rod extruder was utilised, and a lab grade compounding machine was employed. A prep-mixer was used to combine ABS pellets and glass fibre at a rotor speed of 50 rpm at a temperature of 225 °C. Mixtures of 15 and 30 wt% SGF were prepared. Under similar circumstances as the control parameters, a neat ABS has also been developed through the mixer. These mixtures were extruded using a twin screw extrusion unit at 220 °C as preforms. For FDM printing filament, a 1.75 mm diameter cylindrical die was employed. During the process, the barrel temperature ranges between 200 and 230 °C. Figure 4 shows the short length of filaments developed with the assistance of the twin screw extrusion process, which was utilised for the development of the specimen.

### 2.2. Performance Test

A visual macro inspection was carried out to determine whether the developed filament’s dimensional qualities were appropriate for the process of FDM. A digital Vernier calliper was used to conduct various trials at numerous filament positions to determine the filament dimension; 0.1 m of tolerance was applicable to the average filament diameter. Under ideal process circumstances, the short glass fibre (SGF) inclusion resulted in a good mixture with the matrix material, resulting in the filament’s surface diameter being even. The generated filament exhibited no flaws or cracks in the macro examination, and there were no obvious problems in the micro study. Further, there were no visible projections on the filament surface of short glass fibre. In the matrix, the short glass fibre inclusion revealed that the readings of diameter were nearly identical compared to pure ABS, with no influence on the smoothness of the surface.

To comprehend the dimensions, trials have been undertaken at various filament lengths using a digital Vernier calliper. For an average filament diameter value, it had a limiting tolerance of 0.1 mm. With no fluctuation in the necessary diameter, the reinforcement of glass fibre was bonded proportionately to the matrix. Filament’s dimensional accuracy was found to be appropriate which is suggested by the values, which could also be utilised for the process of FDM.

**Figure 5:**
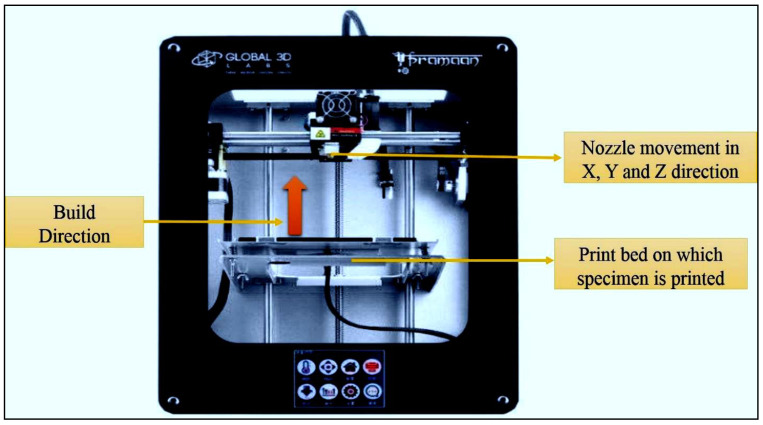
Photograph of FDM machine used for fabrication of parts.

For short glass fibre morphology analysis, we utilised the JSM 7100F Joel model field emission SEM (scanning electron microscope). For the studies of EDAX and SEM experiments, a scanning electron microscope in mode (JSM 840a Jeol) was employed. FDM uses a layer-by-layer approach to model development. For the polymer parts’ development, fused deposition modelling is widely employed, which is a popular 3D manufacturing process. The 3D model is developed from a CAD file, whilst the test specimens for impact and tensile were generated using a Pramaan printer from the laboratories of Global 3D. The printer’s build volume was a 4000 mm^3^ enclosed chamber, and the optimal parameters for the models’ development were chosen. The following parameters were maintained: 100% of infill density, 1.2 mm of top as well as bottom layer thickness, 5 mm/s of speed, 0.1 mm of layer thickness, 0° orientation, and 0.4 mm of shell thickness. The nozzle temperature for printing ABS filament was maintained at 240 °C and was increased for filaments with added SGF reinforcement. The temperature of the bed was maintained at 80 °C. Figure 5 depicts the 3D printer, which is utilised for printing.

For dimensional accuracy, rectangular components of 10 × 10 × 10 mm were developed using FDM. The identical specimens were used for the measurement of surface roughness. Three trials were conducted for length, breadth, and height, and the average value was taken for all axes and compared using a digital Vernier calliper. The instrument was Mitutoyo Corp. (Made in Japan) model number 500-196-200.

With the assistance of a surface roughness tester from Miyu (M35:2010), the Ra (surface roughness) for the FDM parts was measured. A specimen measuring 10 × 10 × 20 mm was developed for the Ra (surface roughness) measurement, on which three trials were conducted at different surface lengths, and the roughness’s average value was examined. The concerned profile’s arithmetic mean deviation was represented by Ra.

**Figure 6:**
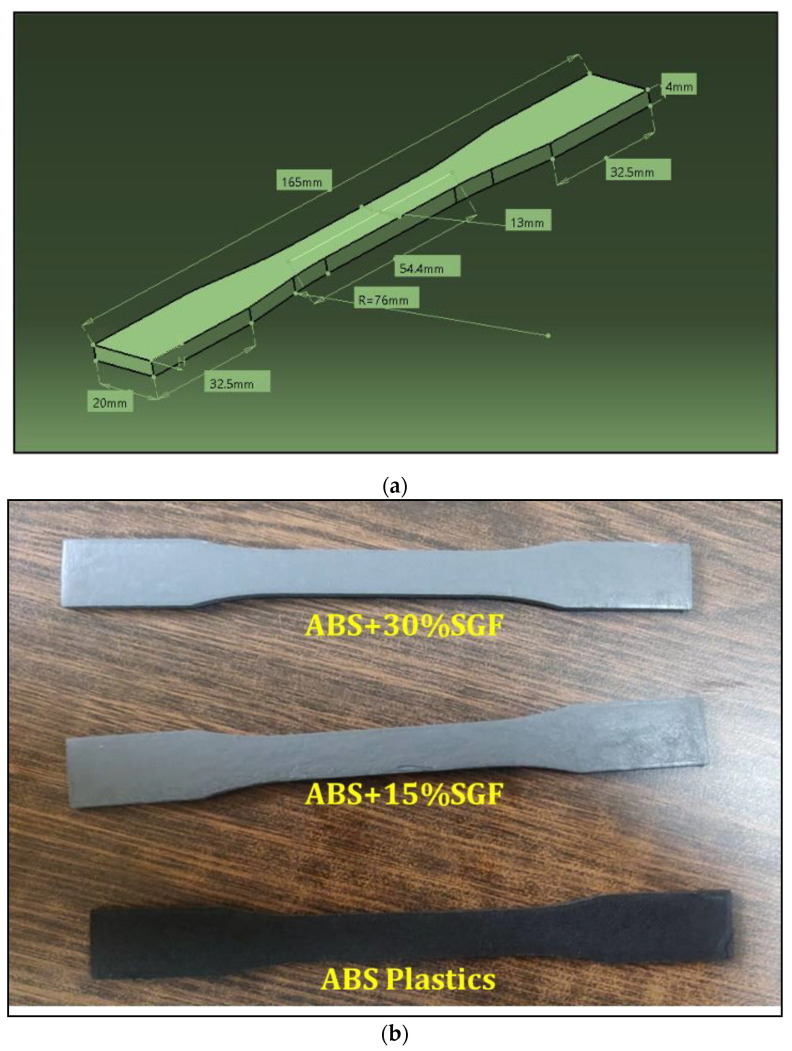
(**a**) Tensile specimen dimensions; (**b**) Photograph of Tensile Specimen.

For the tensile test, the procedure of an ASTM 638 standard approach was employed, and the equipment of FIE (Fuel Instruments and Engineers Private Limited, with a 0–60 tonne capacity machine having been utilised, which passed the tensile test at 2 mm/s speed at room temperature. Every reading of the tensile strength of different composite compositions has been considered as the average of three specimens. The tensile test specimen photograph as well as dog bone model dimensions are shown in Figure 6. The goal of the investigation was to determine the tensile properties of glass fibres as their content increased. To obtain accurate cross section area measurements, entire samples have been measured prior to tensile testing.

The impact test was used to assess the ability of FDM parts to sustain a load as the fibre content increased using an ASTM D256 methodology. The test was conducted using FIE (Fuel Instruments and Engineers Private Limited) equipment along with a 0–60 tonne capacity.

## 3. Results

### 3.1. Microstructure Studies

**Figure 7:**
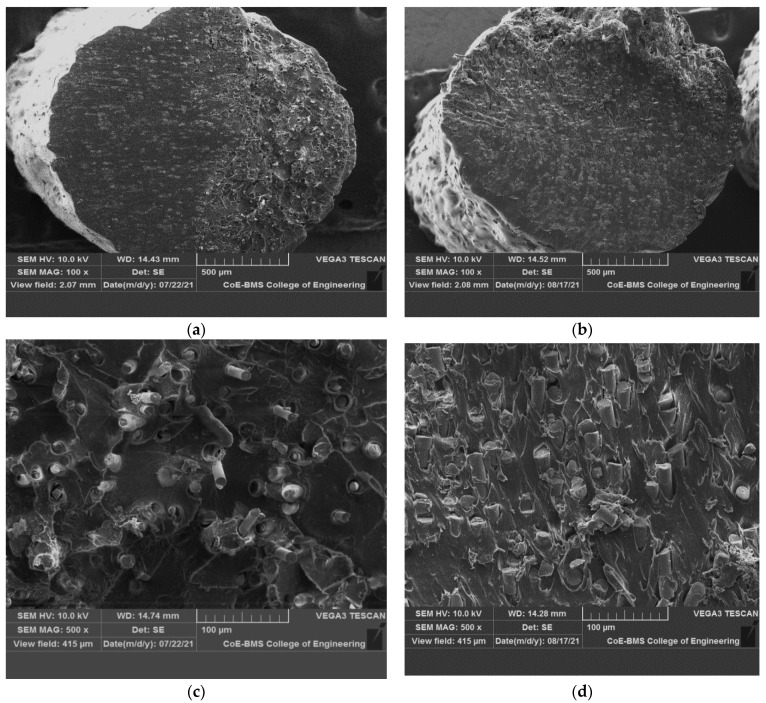

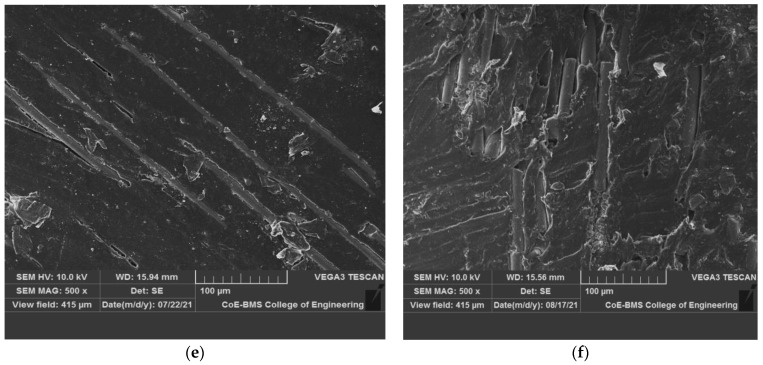
SEM of ABS composite reinforced with different percentage of short glass fibre. (**a**) ABS + 15% SGF; (**b**) ABS+30% SGF; (**c**) ABS + 15% SGF; (**d**) ABS + 30% SGF; (**e**) ABS + 15% SGF; (**f**) ABS + 30% SGF.

Figure 7 depicts the microstructure of ABS and its composites, indicating the uniform distribution of short glass fibres in the matrix without agglomeration. In fibre reinforced composite filament, the better dispersed formation is accomplished by some of the vital factors such as extrusion temperature, compounding temperature, and preheating. It has also been found that the filler’s dispersion properties can be improved by enhancing the content to 30 wt% from 15 wt%, allowing for improved mechanical properties. When a 3D printed part is subjected to external loading, the reinforcing phase carries the majority of the load; however, parameters such as reinforcing phase boding and dispersion may affect load-carrying capacity, and when injected into the ABS matrix, it is expected to help improve the strength. Optimal process conditions for blending in twin screw extruders led to uniform dispersion in 3D printed composites. In FDM, the generated filament utilisation did not result in nozzle blockage up to 30 wt percent glass fibre. In an ABS polymer matrix, the glass fibre dispersion was examined, exhibiting a homogenous distribution of the fibre material with no agglomeration. There is no obvious glass fibre aggregation, as shown in the photographs. Better properties are indicated, and a better surface to volume ratio may be quantified. In the matrix, the GF is found to be bonded. Furthermore, extrusion-induced shear stress caused GF’s alignment along the direction of extrusion [25]; this is because in the polymer matrix, the fibres are extruded as well as the matrix material being constricted, the fibres become aligned with the direction of the nozzle manoeuvre. The increased SGF content after blending did not agglomerate or damage the fibre surface, resulting in improved mechanical properties.

Figure 7c,d shows the SEM image of the extruded filament cut in the filament directions of which clearly demonstrates that the glass fibres are seamlessly embedded in the ABS matrix both in case of 15 wt% GF and 30 wt% GF; this reconfirms the alignment of the glass fibres in the directions of the extrusion during filament development. Similarly, Figure 7e,f shows the images of fibre orientation in the direction parallel to the filament print direction.

### 3.2. Dimensional Accuracy

**Figure 8:**
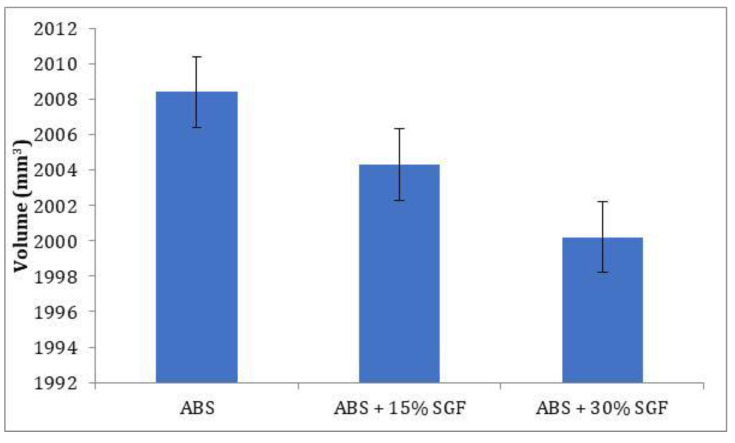
Variation of volume with addition of short glass fibre.

The dimensional accuracy of the developed components was determined by developing a rectangular component. The average values of the developed composites were taken as the final result. The graph in Figure 8 depicts the decrease in dimensional error as the glass fibre content increases. The improvement in dimensional stability is seen with the addition of glass fibre to the ABS matrix. Glass fibre’s high value of thermal conductivity enhances the dimensional accuracy of the parts [26]; here, an important contributor is the coefficient of thermal expansion (CTE). Fillers and fibres with a CTE significantly lower than that of the polymer matrix reduce the CTE of the composite. The interrelationships between polymer-filler differential CTE and induced thermal gradients (both across and along the printing plane) define the distortion and dimensional variations in printed parts [27]. With the addition of 30 wt% glass fibre in the ABS matrix, the maximum dimensional error of the FDM parts is reduced by 0.1% compared to unreinforced ABS.

In the process of FDM, the first layer is laid over the bed of specified thickness, creating a binding zone for the adjacent layer that would be led over it. The optimal temperature maintenance plays a major role in creating diffusion between the layers and allows for rapid cooling due to continual layer deposition. This phenomenon helps in establishing greater bond strength [28]; however, there remains a void between the filaments, which takes the shape of a triangle rather than a circle. The enhanced polymer diffusion with short glass fibre content reduces pore size, leading to stronger bond formation; however, the formation of voids would be very minimal between layers when the fill density is at 100%. The decreased pore size in the case of the glass fibre reinforced ABS composites is due to bond strength enhancement and increased diffusion. The addition of SGF has led to an increase in dimensional stability due to the characteristics of the fibre and also due to the bond strength between ABS and SGF.

### 3.3. Surface Roughness

**Figure 9:**
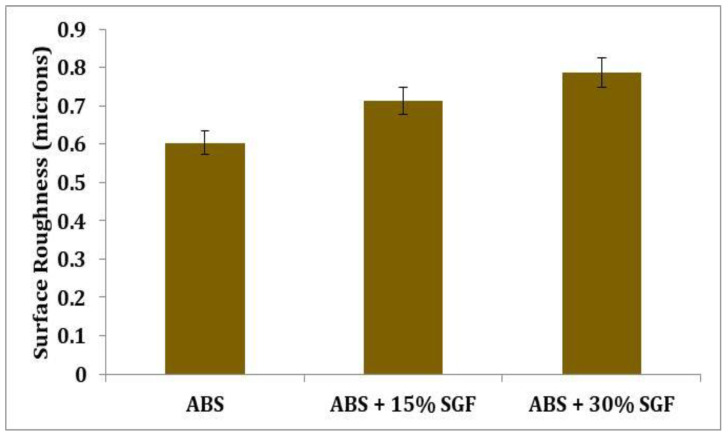
Variation of surface roughness with the addition of short glass fibre.

The average values of roughness (Ra) are presented in the graph, which is shown in Figure 9 to assess the roughness between various specimens. The graph depicts the increase in surface roughness as short glass fibre content is increased; indeed, the glass fibre reinforcement inclusion might be responsible for the deterioration of the surface finish. Surface roughness is increased by 18.1% when compared to pure ABS with a 15% weight-addition of glass fibre; likewise, with the reinforcement inclusion, the increasing trend is also maintained. When compared to the glass fibre of 15 wt%, the glass fibre of 30 wt% inclusion-ABS resulted in a 12.1% reduction.

### 3.4. Mechanical Properties

#### 3.4.1. Tensile Properties

**Figure 10:**
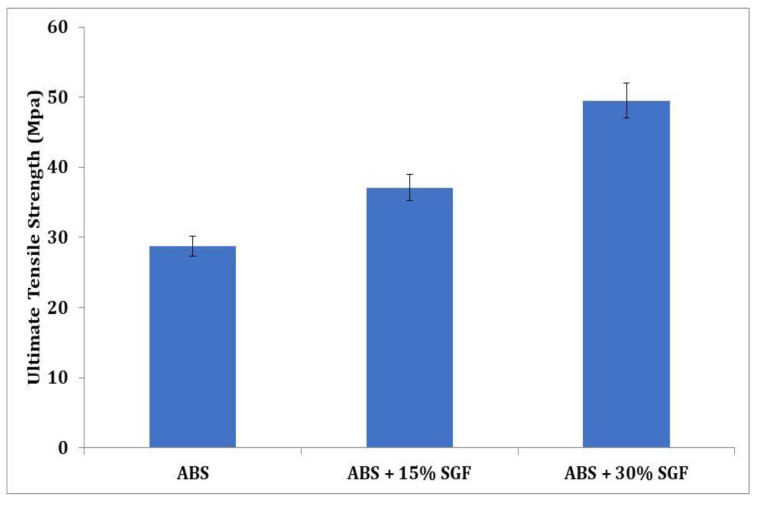
Variation of tensile strength with increase in SGF content.

Figure 10 depicts the variances in ultimate tensile strength for ABS–SGF composites as a short glass fibre content function. The inclusion of glass fibre effectively increases ultimate strength. Compared to the ABS-15 wt% short glass fibre composites, the ABS-30 wt% short glass fibre composites’ strength is greater, which might be observed. Additionally, it is found that the composite strength improves noticeably when the percentage of fibre weight increases. When compared to both materials, the increase in UTS is quite significant. Strictly speaking, the addition of SGF has resulted in a significant increase in the UTS of the ABS matrix; this is primarily due to the reinforcing phase’s primary role in carrying the majority of the load when subjected to external loading conditions. The efficiency of the reinforcing phase’s load-carrying capacity is affected by a variety of factors, such as the wetting of the reinforcement with the matrix, dispersion of the reinforcing phase, and reinforcement content. One can see that the strength of SGF is quite high, and it is expected that when it is introduced into the ABS matrix, it will help to improve the strength; furthermore, the decomposition temperature and chemical stability of SGF are exceptional, and they can be expected to retain their dimensions and strength at elevated temperatures as well. This is in contrast to other reinforcements, which tend to decompose during the blending process in twin screw extruders or agglomerate in 3D printed composites. As the SGF content was increased from 15% to 30%, the load-carrying capacity of the ABS composite increased; the load-carrying capacity of the composite is reduced at a low SGF content of 15%, but when it is increased to 30%, the number of load-carrying fibres increases, allowing the composite to carry more loads than an ABS/15% SGF composite. One cannot simply assert that increasing the SGF content will increase UTS, because the higher the reinforcement content, the more problems with dispersion and bonding will arise; however, good interfacial bonding and uniform dispersion of SGF were observed in both the filament and the 3D printed part. Despite the increased SGF content, no agglomeration or damage to the fibre surface was observed after the blending process. Even after the printing process, the part surface showed no broken fibres protruding from its surface or any significant pores that could have formed as a result of poor mixing or printing parameters; the interfacial bonding was also excellent, with no gaps observed at the interface between the SGF and the ABS matrix. This implies that the SGF was densely compacted in the ABS matrix, and that such a structure can accommodate large strains whilst preventing crack initiation. As a result of these factors, the ABS/30% SGF composite had a higher UTS value.

**Figure 11:**
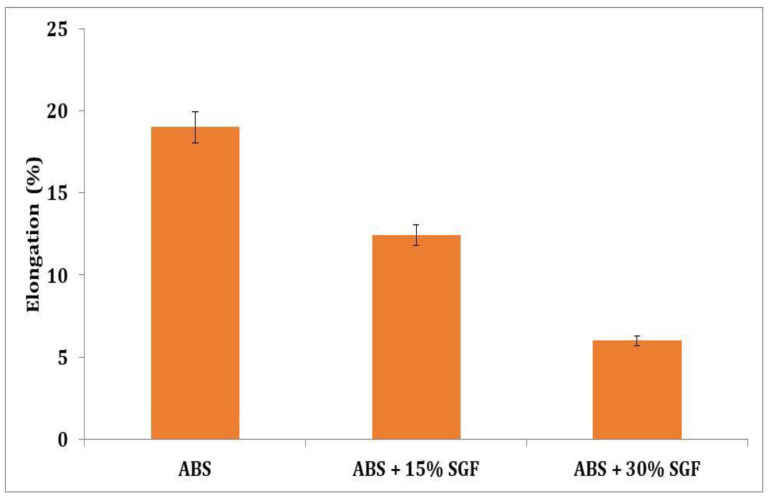
Variation of percentage elongation with short glass fibre content.

Figure 11 indicates the reduction in elongation with the increased addition of SGF. When 15 wt% SGF was added to pure ABS, the percentage of elongation was reduced by 46%. The 30 wt% addition of SGF resulted in a further reduction in elongation. The polymer matrix gave enhanced results with an increment of SGF; however, the reduction in elongation was noted.

**Figure 12:**
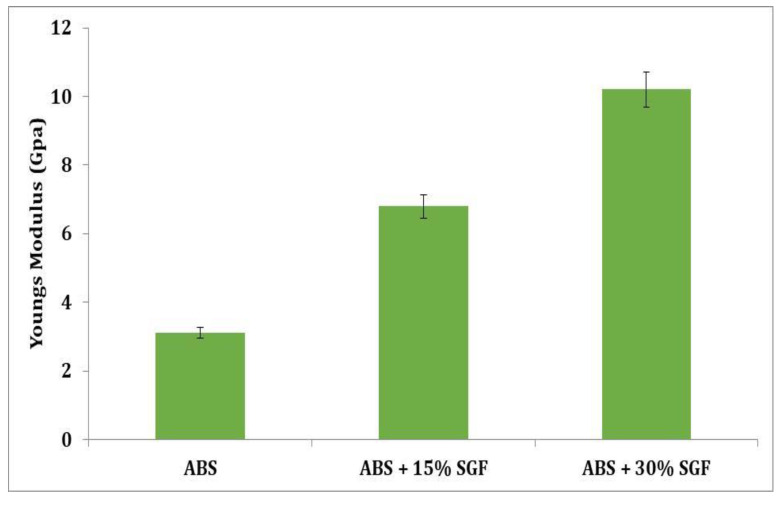
Variation of Young’s modulus with short glass fibre content.

Figure 12 depicts the variations in ABS elastic modulus as a consequence of fibre volume fraction. It is observed that as the fibre concentration increases, both the composites’ modulus also increase considerably; the fibre orientation changes can thus be ignored because in all the specimens the fibres are aligned preferentially alongside the direction of flow. At low strain, the modulus is a material property, and one that is highly delicate towards the interface of the fibre matrix.

Despite the gaps, the printed samples had better tensile strength, indicating that the process of FDM enhances the polymer chains’ molecular orientation, improving the properties of tensile [27]. Because the products are made layer-by-layer and point by point, the FDM technique not only enhances the polymer molecules’ orientation, but it also improves homogeneity as well as fibre dispersion.

As per the outcomes, using the layer thickness of 0.1 mm (the thinnest available in the current investigation) leads to an increased strength of the tensile, perhaps by improving interlayer adhesion [29,30,31].

GF incorporation enhances elastic modulus by 14% and 26% for ABS + 15 wt% short glass fibre and ABS + 30 wt% short glass fibre composites, respectively, in comparison to those of pure ABS; this might be due to the rise in ABS/glass fibre interphase stiffness. A flawless interface implies a greater modulus for the composite due to a faster rate of stress transmission through the interface of fibre or matrix contact. Furthermore, when compared to pure ABS, the tensile strength of ABS + 15 wt% short glass fibre (14%) and ABS + 30 wt% short glass fibre composites increased. Such increases indicate improved stress transfer and greater inference of stronger interfacial interactions between the ABS or fibre interface. At break, the strain followed the same pattern as the strength, in which at break the strain in ABS + 15 wt% short glass fibre and ABS + 30 wt% short glass fibre composites decreased by 14 and 10%, respectively, compared to pure ABS. Furthermore, because these qualities are often dominated by flaws within the composite, a distinct mechanism could be at work for strain as well as strength at break. At break properties, the strain and the strength of materials having defect mediated failure will depend on the number of flaws as well as on their size in relation to the sample’s high-stressed areas and the volume being put through the greatest tensile stress.

#### 3.4.2. Fractures Specimen Analysis

**Figure 13:**
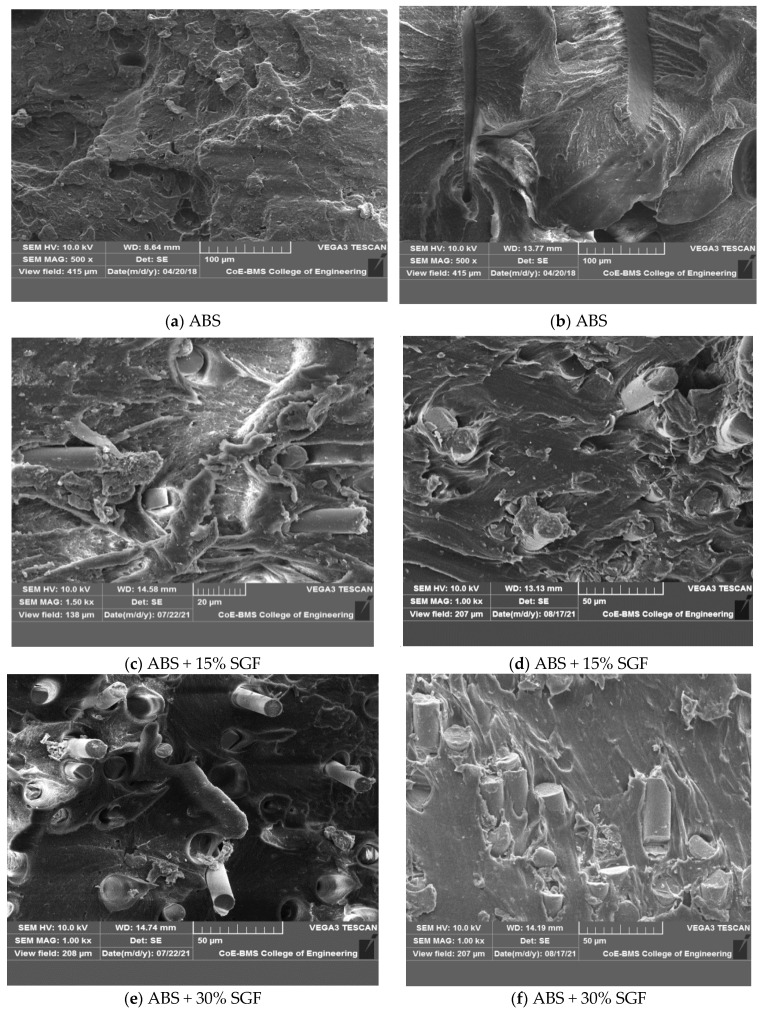
SEM of fractured tensile specimens of ABS, ABS + 15 wt% Glass fibre and ABS + 30 wt% glass fibre.

Figure 13 shows ABS and various glass fibre weight percentages included in the tensile specimens of composites with microscopic fracture surfaces. Because of normal stress, the failure of composite specimens was kept at right angles in-plane to the cross section of the plane, and it was connected to the certainty of high stiffness as well as perfect brittleness. The composite specimen’s mode of failure, on the other hand, was similar to that of the type of unclad composite specimen, as shown in Figure 13c–f; in the end, it departed with a short fibrous fracture. In contrast, failure had occurred in the plane angled to the cross section of the plane because of shear stress in composite specimens with glass fibre’s higher percentage; as a result, there is a lengthy damaged length as well as a lengthy fibrous fracture at the conclusion. From the matrix to fibre, any load cannot be transferred by the debonded interface, and debonding length is utilised to predict interfacial energy as well as fibre stress.

In reinforced composites, a brittle matrix fracture was noticed. Throughout the thickness of the specimen, the fibres were aligned selectively along the direction of flow. A SEM was used to investigate the fractured surface of pure ABS and glass fibre reinforced composites that ended in failure whilst testing of the tensile. Interfacial debonding, fibre breakages as well as concurrent matrix cracking were the predominant failure mechanisms for the composites, as illustrated in Figure 13c–f, and this suggests that the interfacial bonding has improved. For both ABS + 30wt% SGF composites as well as ABS + 15 wt% SGF, a rough fracture surface on the pulled-out fibres from the matrix residues imply greater fibre matrix adhesion.

The fracture surfaces of tensile fractured specimens were practically normal to the direction of loading, indicating macroscopic failure. The surface of the fracture had been planar, perpendicular to the direction of loading, as well as positioned between the adjacent filaments at the interface.

In glass fibre reinforced specimens’ case, the fibre pull-out as well as fibre fracture had been the most common damaging mechanisms; moreover, the filaments, and therefore the fibres, are oriented positively, locally in regard towards the applied stresses as a result of this infill method. This research stresses the significant link between the geometry of 3D printed objects as well as considering the influence of the production process on the final mechanical qualities. As seen in Figure 13, both composite specimens had a small number of broken fibres; indeed, earlier macroscopic investigation revealed that the crack spread between adjacent filaments at the interface, and between layers the delamination’s almost non-existent.

It is necessary to improve the interfacial bonding between the fibre and the matrix in order to increase the functional performance of 3D printed polymer composites, which improve the performance, whilst improving the performance of 3D printed polymer composites may also be achieved by reducing the amount of porosity present; further, as part of future scope of the study, the effect of parameters such as fibre volume fraction, ambient environment, cooling rate, nozzle temperature, and printing speed in order to reduce porosities should be explored.

FRP composites that have been produced in three dimensions have a substantial influence on the fatigue performance of the composites that have been 3D-printed. It is feasible to get increased fatigue strength by raising the volume percentage in the mixture; an increase in fibre orientation might result in a decrease in fatigue resistance, which is something that has to be investigated in greater depth as part of future research.

## 4. Conclusions

The present research article focused on the development of ABS-reinforced SGF using the process of fused deposition modelling. The filaments of 1.75 mm in diameter were developed from pure ABS and ABS-reinforced SGF. The proportions of SGF added were 15 and 30 wt%. The filaments were developed using the twin screw extrusion process. The extraction of filaments did not create any clogs in the screws, which led to the filaments being successfully extracted.

The results of the printed samples showed greater values of mechanical and physical properties than the samples printed with fibre orientation in the print direction. The addition of SGF has increased the Young’s modulus and tensile strength of the samples; however, the decrement was observed in elongation with the incremental addition of SGF, which suggests the material is moving towards a brittle nature. The microstructure studies of fractured specimens indicated the breaking of fibres instead of a complete pull-out from the matrix, a phenomenon which suggests a strong interface between the matrix and fibre. The fibre tries to withhold the matrix to its maximum extent of load, resulting in increased strength.

In conclusion, this research article suggested a method for the development of novel materials using the process of FDM. Better dispersion capabilities were achieved, reduced voids were noted with the incremental addition of SGF, and clogging of the nozzle was not seen. This paves the path for the development of high-strength polymer composites using FDM.

## Data Availability

The data presented in this study are available on request from the corresponding author.
